# The fatty liver index as a predictor of incident chronic kidney disease in a 10-year prospective cohort study

**DOI:** 10.1371/journal.pone.0180951

**Published:** 2017-07-24

**Authors:** Ji Hye Huh, Jang Young Kim, Eunhee Choi, Jae Seok Kim, Yoosoo Chang, Ki-Chul Sung

**Affiliations:** 1 Department of Internal Medicine, Yonsei University, Wonju College of Medicine, Wonju, Korea; 2 Biostatistician, Smith Center for Outcomes Research in Cardiology, Beth Israel Deaconess Medical Center, Boston, United States of America; 3 Department of Occupational and Environmental Medicine, Kangbuk Samsung Hospital, Sungkyunkwan University, School of Medicine, Seoul, Korea; 4 Division of Cardiology, Department of Medicine, Kangbuk Samsung Hospital, Sungkyunkwan University School of Medicine, Seoul, Korea; The University of Tokyo, JAPAN

## Abstract

**Background:**

Although non-alcoholic fatty liver disease (NAFLD) is considered to be associated with chronic kidney disease (CKD), long-term follow up data is lacking. We investigated whether NAFLD, as determined by the fatty liver index (FLI), could predict incident CKD in 10-year prospective cohort study. We also assessed the clinical utility of FLI to predict the development of CKD.

**Methods:**

6,238 adults aged 40 to 69 years without baseline CKD from the Ansan—Ansung cohort were examined. Patients were classified according to FLI as follows: FLI<30, no NAFLD; FLI≥60, NAFLD; and 30≤ FLI<60, intermediate. Incident CKD was defined as estimated glomerular filtration rate (eGFR) <60 ml/min per 1.73 m^2^. The clinical utility of FLI in predicting incident CKD was estimated via area under the receiver-operating characteristic curve (AUC), net reclassification improvement (NRI), and integrated discrimination improvement (IDI) analyses.

**Results:**

During an average of 10 years of follow-up, 724 subjects (15.21%) developed CKD. The adjusted hazard ratio [95% confidence interval (CI)] for incident CKD increased in a graded manner with FLI increased (<30 vs. 30–59 vs. ≥60 = 1 vs. 1.17 [0.997–1.375] vs. 1.459 [1.189–1.791], respectively, P for trend = 0.0012). Incorporation of FLI into traditional risk factors of CKD significantly increased prediction of incident CKD based on NRI (17%; 95% CI, 8.9–25%; P-value <0.001) and IDI (0.002; 95% CI, 0.0046–0.0143; P-value = 0.046).

**Conclusions:**

FLI, a surrogate marker of NAFLD, was an independent risk factor for incident CKD. FLI provides meaningful incremental risk reclassification beyond that of conventional risk factors of CKD.

## Introduction

Chronic kidney disease (CKD) has become a worldwide health problem that results in high morbidity and mortality in various chronic diseases, consuming substantial healthcare costs. The prevalence of CKD in developed countries has been reported as approximately 10–15% of the adult population and is expected to rise in the future as populations age and the prevalence of obesity and diabetes mellitus increase [1–3]. Recently, CKD has come to be considered a risk factor for not only end-stage renal disease, but also cardiovascular disease, even in the early stages of renal dysfunction. Furthermore, a large body of data indicates that CKD is associated with all-cause mortality, premature death, cognitive impairment, and poor quality of life. Therefore, it is important to identify other modifiable risk factors of CKD in addition to traditional risk factors such as obesity, diabetes mellitus, and hypertension.

Non-alcoholic fatty liver disease (NAFLD) is the most common cause of chronic liver disease, with a prevalence as high as 30% of the general population in developed countries [4]. The definition of NAFLD is the accumulation of fat (>5%) in liver cells in the absence of excessive alcohol intake or other causes of liver disease [5]. NAFLD comprises a disease spectrum ranging from simple steatosis progressing through non-alcoholic steatohepatitis (NASH) with liver fibrosis, often followed by liver-related complications such as liver failure or hepatocellular carcinoma [6]. NAFLD is considered to be a risk factor for type 2 diabetes, insulin resistance, and cardiovascular disease [7,8], and there is growing evidence to support it as a cause of incident CKD [9].

NAFLD and CKD may share many important common cardio-metabolic risk factors such as insulin resistance, chronic inflammation, and obesity. In addition, several cross-sectional studies have demonstrated that NAFLD assessed by ultrasonography is associated with increased risk of prevalent CKD [10,11]. This association raises the possibility that the link between NAFLD and CKD might not simply be mediated by shared, underlying, common risk factors, but rather that NAFLD independently contributes to increasing this risk. However, there were few data assessing the impact of hepatic steatosis on development of CKD in a large-scale long-term follow up study, independent of common risk factors among these two distinct diseases. In addition, while an increased prevalence of CKD in NAFLD is largely accepted, there is still very little prospective data linking NAFLD to decline of renal function.

Although the most common technique used to assess the presence of NAFLD is ultrasonography, this test has limitations for the detection of milder degrees of steatosis (<33% fat in hepatocytes), which requires a liver biopsy [12,13]. Bedogni et al. developed a simple scoring system called the fatty liver index (FLI) as a predictor of hepatic steatosis [14], and it has been well validated in Korean populations and other ethnic groups [15,16]. The objective of this study was determining the independent association between hepatic steatosis as measured by FLI and new-onset CKD over a 10-year follow-up period. We also assessed the clinical utility of FLI for predicting incident CKD in a large-scale community-dwelling population.

## Materials and methods

### Study population and design

**Fig 1:**
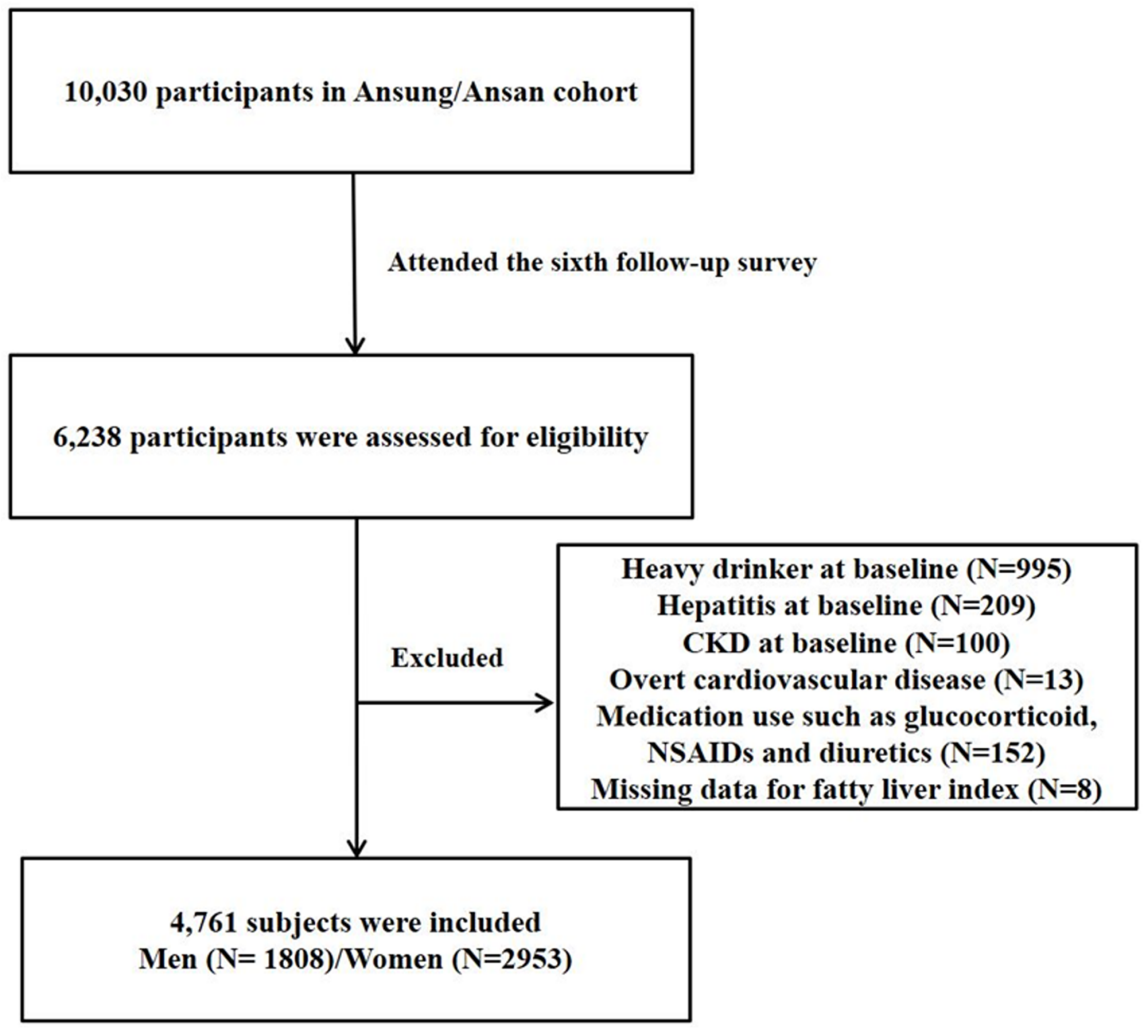
Study populations. CKD, chronic kidney disease; NSAIDs, nonsteroidal anti-inflammatory drugs.

We used data from the Ansung-Ansan cohort study, which is an ongoing prospective study starting in 2001 embedded within the Korean Genome and Epidemiology Study (KoGES), a population-based prospective cohort study, to assess the prevalence, incidence, and risk factors for chronic degenerative disorders such as hypertension, diabetes, and cardiovascular disease [17] [18]. The Ansung cohort represents a rural community, and the Ansan cohort is an urban community. Each cohort consists of a population-sample of Korean men and women aged 40–69 years with the same ethnic background. The baseline survey, carried out from 2001 to 2002, included 10,030 adults, who were examined again at 2-year intervals; ultimately 6,238 subjects participated in the sixth follow-up survey. Of these participants, 1,477 were excluded for one or more of the following reasons: GFR < 60 ml/min per 1.73 m2 at the baseline examination (N = 100); missing data for the FLI (N = 8); history of symptomatic cardiovascular disease (N = 13); history of hepatitis (N = 209); excessive alcohol intake (alcohol consumption >140 g/week for men and > 70 g/week for women) (N = 995); use medications associated with renal function decline or fatty liver within the past 3 months, such as glucocorticoids, nonsteroidal anti-inflammatory drugs (NSAIDs), or diuretics (N = 152). In the end, 4761 participants (1808 men and 2953 women) were recruited for the present study (Fig 1).

At each visit, informed written consent was obtained from all participants. The study protocol was approved by the Ethics Committee of the Korean Center for Disease Control and the Institutional Review Boards of the Yonsei University Wonju College of Medicine (YWMR-15-9-075). This study was carried out in accordance with the ethical standards of the Helsinki Declaration.

### Clinical and laboratory measurements

All participants completed a comprehensive health examination and interviews according to a site visit schedule. The health examination included an evaluation of anthropometric indices and the collection of biological specimens for assessment. Waist circumference was measured in a horizontal plane midway between the inferior margin of the ribs and the superior border of the iliac crest. Participants also completed interviewer-administered questionnaires on medical history including medication use, family disease history, and lifestyle factors such as smoking status and alcohol intake. Physical activity was classified into none/irregular (≤ 2 episodes/week) or regular (≥ 3 episodes/week) exercise. One episode of exercise was defined as exercising for at least 30 min. Blood pressure was measured by trained technicians using mercury sphygmomanometers (Baumanometer-Standby; W.A. Baum Co. Inc., New York, NY, USA). The mean of two blood pressure readings was used for data analyses. After overnight fasting for 12 h, the plasma concentrations of glucose, γ-glutamyltransferase (GGT), total cholesterol, triglycerides (TG), high-density lipoprotein (HDL) cholesterol, and low-density lipoprotein (LDL) cholesterol were measured enzymatically using a 747 Chemistry Analyzer (Hitachi, Tokyo, Japan). Plasma insulin concentrations were determined using a radioimmunoassay kit (Linco Research, St. Charles, MO, USA). Serum creatinine was measured using Jaffe’s method with a Hitachi Automatic Analyzer 7600 (Hitachi, Tokyo, Japan). The HOMA-IR index was calculated as fasting plasma glucose (milligrams per deciliter) ×fasting insulin (milli-international units per milliliter))/22.5 [19].

### Definition of incident CKD

Estimated glomerular filtration rate (eGFR) was calculated using the CKD-Epidemiology Collaboration (CKD-EPI) equation [20], eGFR (mL/min per 1.73 m^2^) = 141 × min(Scr/κ, 1)^α^ × max(Scr/ κ,1)^-1.209^ × 0.993^age (years)^ × 1.018 (if female) × 1.159 (if black), where κ is 0.7 for females and 0.9 for males, α is –0.329 for females and–0.411 for males, min indicates the minimum of Scr/κ or 1, and max indicates the maximum of Scr/κ or 1. The definition of incident CKD was an eGFR < 60 ml/min per 1.73 m^2^ with a baseline eGFR ≥ 60 ml/min per 1.73 m^2^ at six consecutive follow up visits [21].

### Measurement of fatty liver index and definition of NAFLD

A well validated and surrogate marker, the FLI was used to identify patients with NAFLD. FLI was calculated according to a previously published report by Bedogni et al. [14]: FLI = [e ^0.953^×log_e_ (TG) + 0.139×BMI+0.718×log_e_ (GGT) +0.053×waist circumference–15.745)] / [1+e^0.953^× log_e_ (TG) + 0.139×BMI+0.718×log_e_ (GGT) + 0.053×waist circumference–15.745] × 100, with TG measured in mmol/l, GGT in U/l, and waist circumference in cm. In accordance with the previous reports, we classified participants into three groups according to the FLI values: FLI <30 was suggested rule out NAFLD, FLI ≥60 was suggested rule in NAFLD, and FLI 30–59 as intermediate.

### Statistical analysis

Statistical analyses were performed using SAS 9.4 Ver. (SAS Institute Inc., Cary, NC, USA). Continuous variables are presented as means and standard deviations. Categorical variables were expressed as frequencies and percentages. The characteristics of the study population among each group were compared using one-way analysis of variance (ANOVA) and two-sample t-test for continuous variables, or Chi-square test for categorical variables. Cox’s proportional hazards model was applied to determine the independent association between FLI and incident CKD after adjustment for confounding variables. The cumulative incidence of CKD by FLI group was assessed by Kaplan–Meier plots. Results were expressed as hazard ratios with 95% confidence intervals (CI). Finally, we compared the traditional and new model including FLI using the same area under the curve (AUC) comparison as DeLong [22]. We used net reclassification improvement (NRI) with a category-free option and integrated discrimination improvement (IDI) calculations to quantify the improvement in actual reclassification and sensitivity based on the addition of FLI to the traditional existing model [23]. P values less than 0.05 were considered statistically significant.

## Results

### Baseline characteristics of the study subjects

Baseline characteristics of participants based on the development of CKD over the 10-year follow up period are shown in Table 1. Among the 4,761 subjects, 15.21% (N = 724) developed CKD during the follow-up period. Incident CKD group had significantly higher baseline age, waist circumference, BMI, blood pressure, total cholesterol, LDL-cholesterol, TG, and hsCRP levels than non-incident CKD group. There were not significant differences in AST, ALT, and GGT levels at baseline among the incident and non-incident CKD groups. Baseline FLI levels were higher in the incident CKD group than in the non-incident CKD group (33.66±23.92 vs. 28.03±22.39, P<0.001).

**Table 1:** Baseline characteristics according to incident CKD.

Baseline variables	Incident CKD(+) N = 724 (15.21%)	Incident CKD(-) N = 4037 (84.79%)	p-value
Age(years)	58.74±8.00	50.72±8.03	< .0001
Sex (men)	254 (35.08%)	1554 (38.49%)	0.08
BMI (kg/m^2^)	24.87±3.27	24.53±3.05	0.01
Waist circumference (cm)	84.43±9.07	81.74±8.75	< .0001
HTN	258 (37.55%)	796 (20.82%)	< .0001
Systolic BP (mmHg)	126.4±19.80	119.3±17.50	< .0001
Diastolic BP (mmHg)	81.55±11.08	79.06±11.09	< .0001
Diabetes mellitus	86 (11.96%)	171 (4.26%)	< .0001
HbA1c (%)	6.08±1.35	5.69±0.72	< .0001
FPG (mg/dL)	89.43±26.67	84.79±16.74	< .0001
Total cholesterol (mg/dL)	196.9±35.96	188.7±33.68	< .0001
HDL- cholesterol (mg/dL)	43.41±9.55	44.41±9.72	0.01
LDL-cholesterol (mg/dL)	119.0±32.13	113.8±31.76	< .0001
Triglyceride (mg/dL)	172.2±108.6	152.4±92.91	< .0001
e-GFR (mL/min per 1.73 m^2^)	83.60±12.27	95.45±12.20	< .0001
HOMA-IR	1.78±1.30	1.62±1.08	0.003
AST (IU/L)	28.33±12.13	27.99±17.23	0.52
ALT (IU/L)	25.98±18.39	26.41±31.08	0.61
GGT (IU/L)	29.55±81.19	24.62±29.18	0.11
FLI	33.66±23.92	28.03±22.39	< .0001
Albumin (g/dL)	4.23±0.29	4.23±0.31	0.70
hsCRP (mg/dL)	0.26±0.35	0.21±0.59	0.007
Current smoking	125 (17.27%)	750 (18.58%)	0.54
Current alcohol intake	229 (31.98%)	1555 (38.90%)	0.0008
Regular exercise (none)	231 (32.31%)	1221 (30.81%)	0.43
Protein intake (g/day)	59.89±26.27	66.49±29.22	< .0001

CKD, chronic kidney disease; BMI, body mass index; BP, blood pressure; HbA1c, hemoglobin A1c; FPG, fasting plasma glucose; HDL, high-density lipoprotein; LDL, low-density lipoprotein; eGFR, estimated glomerular filtration rate;HOMA-IR, homeostatic model assessment of insulin resistance;; AST, aspartate aminotransferase; ALT, alanine aminotransferase;GGT, γ-glutamyltransferase;hsCRP, high-sensitivity C-reactive protein

Table 2. presents the characteristics of study participants according to baseline FLI levels. A total of 601 (12.62%) subjects had NAFLD as assessed by FLI (≥60), and 2889 (60.68%) subjects did not have NAFLD (FLI<30). Subjects with higher FLI had lower eGFR at baseline.

**Table 2:** Baseline characteristics according to FLI category.

Baseline variables	FLI			p-value
	<30 N = 2889 (60.68%)	30~59 N = 1271(26.70%)	≥60 N = 601(12.62%)	
Age(years)	51.24±8.58	53.24±8.36	52.54±8.23	< .0001
Sex (men)	899 (31.12%)	564 (44.37%)	345 (57.40%)	< .0001
BMI (kg/m^2^)	23.09±2.35	26.22±2.30	28.30±2.78	< .0001
Waist circumference (cm)	77.53±6.78	87.37±5.83	93.29±6.57	< .0001
HTN	431 (15.48%)	384 (32.85%)	239 (42.83%)	< .0001
Systolic BP (mmHg)	116.75±17.63	124.44±16.88	128.97±17.70	< .0001
Diastolic BP (mmHg)	76.93±10.73	82.40±10.56	85.23±10.45	< .0001
Diabetes mellitus	81 (2.82%)	100 (7.91%)	76 (12.75%)	< .0001
HbA1c (%)	5.59±0.68	5.89±0.95	6.17±1.17	< .0001
FPG (mg/dL)	82.94±14.82	87.42±19.70	93.98±27.92	< .0001
Total cholesterol (mg/dL)	184.22±32.42	195.67±32.82	205.29±38.12	< .0001
HDL- cholesterol (mg/dL)	46.46±9.72	41.39±8.72	39.73±8.35	< .0001
LDL-cholesterol (mg/dL)	114.15±28.90	117.41±32.12	110.50±39.22	< .0001
Triglyceride (mg/dL)	118.04±46.37	184.39±81.98	275.30±158.74	< .0001
e-GFR (mL/min per 1.73 m^2^)	94.62±13.00	92.37±12.39	91.70±13.27	< .0001
HOMA-IR	1.43±0.85	1.85±1.26	2.31±1.55	< .0001
AST (IU/L)	25.89±8.17	28.76±13.39	36.85±37.06	< .0001
ALT (IU/L)	20.94±9.72	29.91±28.43	44.78±65.07	< .0001
Total bilirubin (mg/dL)	0.58±0.30	0.56±0.27	0.61±0.36	0.005
GGT (IU/L)	15.42±9.79	29.79±26.66	63.86±98.64	< .0001
Albumin (g/dL)	4.21±0.30	4.24±0.30	4.28±0.31	< .0001
hsCRP (mg/dL)	0.19±0.59	0.24±0.52	0.31±0.45	< .0001
Current smoking	441 (15.26%)	261 (20.54%)	173 (28.79%)	< .0001
Current alcohol intake	1007 (35.26%)	486 (38.51%)	291 (48.91%)	< .0001
Regular exercise (none)	909 (32.05%)	377 (30.14%)	166 (28.09%)	0.12
Protein intake (g/day)	65.19±29.12	64.94±27.61	68.15±30.29	0.06

CKD, chronic kidney disease; BMI, body mass index; BP, blood pressure; HbA1c, hemoglobin A1c; FPG, fasting plasma glucose; HDL, high-density lipoprotein; LDL, low-density lipoprotein; eGFR, estimated glomerular filtration rate;HOMA-IR, homeostatic model assessment of insulin resistance;; AST, aspartate aminotransferase; ALT, alanine aminotransferase; GGT, γ-glutamyltransferase; hsCRP, high-sensitivity C-reactive protein

### Risk of incident chronic kidney disease according to baseline FLI groups

Table 3 shows the risk of incident CKD according to the baseline FLI groups. With the FLI<30 group as the reference, the hazard ratios for incident CKD increased across FLI groups. After adjustment for confounding variables, this trend remained statistically significant (Model 2). In a fully adjusted model (Model 3), the hazard ratios (95% CI) for incident CKD in the 30≤FLI≤59 and FLI ≥60 groups were 1.17 (0.997–1.375) and 1.459 (1.189–1.791) compared to those in the FLI<30 group (P for trend = 0.0012).

**Table 3:** Hazard ratios (95% Confidence interval) for incident CKD according to the FLI groups.

	FLI			P for trend
	<30	30~59	≥60	
Incident CKD case	379 (13.12%)	224 (17.62%)	121 (20.13%)	<0.001
Crude hazard ratios	1	1.418 (1.221~1.648)	1.723 (1.434~2.070)	<0.0001
Model 1	1	1.260 (1.084~1.464)	1.690 (1.405~2.032)	<0.0001
Model 2	1	1.175 (1.002~1.377)	1.468 (1.205~1.789)	0.0006
Model 3	1	1.170 (0.997~1.375)	1.459 (1.189~1.791)	0.0012

Model 1: Adjusted for age and sex. Model 2: Model 1 + further adjusted for baseline eGFR, smoking, regular exercise, alcohol intake, protein intake, systolic BP and DM. Model 3: Model 2 + further adjusted for total cholesterol and log hsCRP.

**Fig 2:**
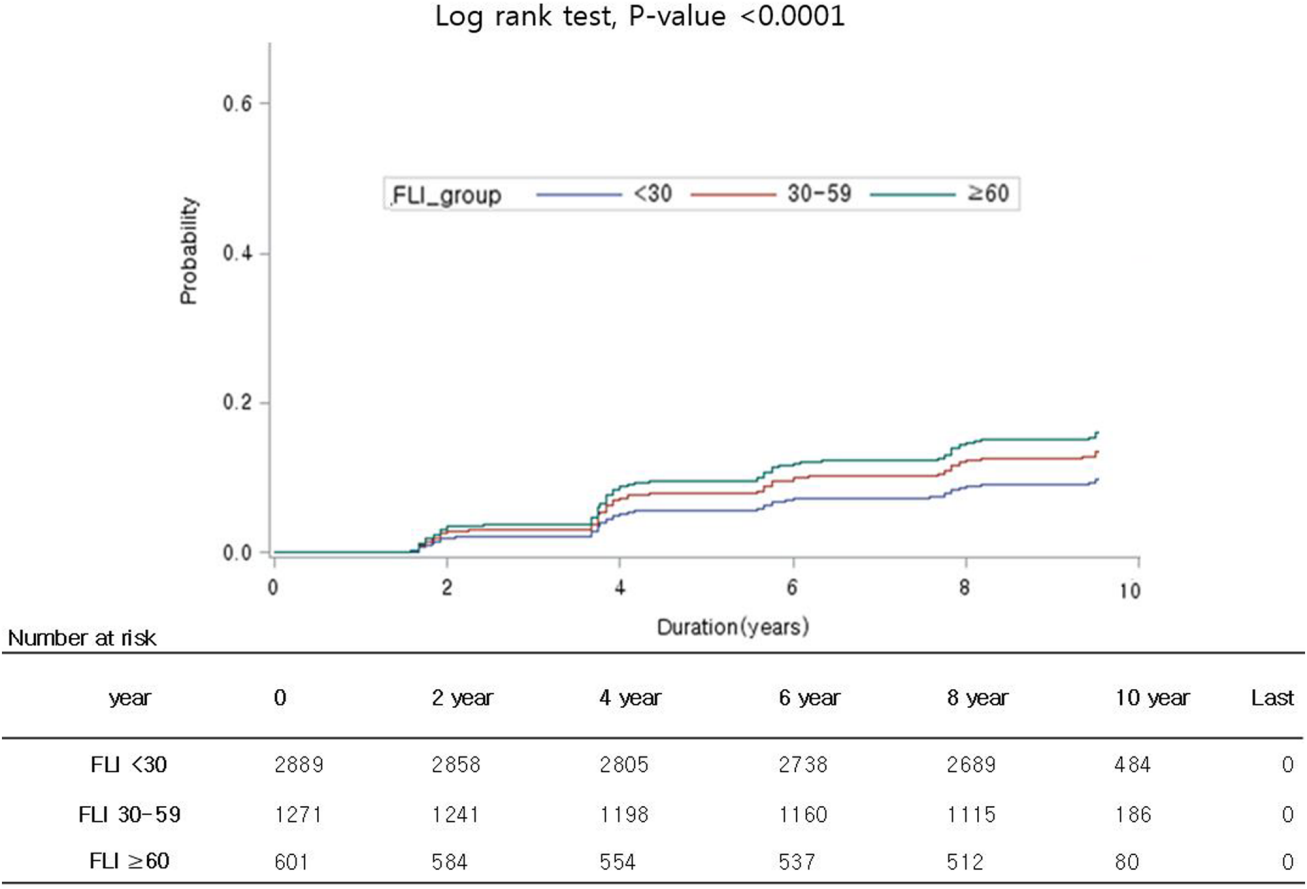
Kaplan-Meier curves of incident CKD according to FLI categories. CKD, chronic kidney disease; FLI, fatty liver index.

As expected, the risk of incident CKD was higher in higher FLI group and the increased CKD risk in higher FLI group persisted up to the 10-year follow-up (log-rank test, P-value < 0.001) (Fig 2).

### FLI in the prediction of incident CKD

We next evaluated the incremental predictive value of FLI when added to a base model of established clinical CKD risk factors. The addition of FLI to a clinical model predicting incident CKD resulted in a modest increase in the AUC (from 0.816 to 0.818, P = 0.0615) (Table 4). Incorporation of FLI also led to significant improvements in reclassification [category-free NRI, 17%; 95% CI, 8.9–25%]. The category-free NRI for event components was -8% (P-value = 0.0359), and that for non-events was 25% (P-value <0.001). This finding indicates that FLI correctly reclassified subjects without events as lower risk. The addition of FLI to the traditional CKD model improved the IDI significantly (IDI, 0.002; 95% CI, 0.0046–0.0143; P-value = 0.0466).

**Table 4:** Discrimination and reclassification improvement for incident CKD by FLI.

	Incident CKD	
	Values	P-value
**AUC (95% CI)**		
Traditional model^a^	0.816 (0.80~0.833)	
Traditional model^a^+FLI	0.818 (0.802~0.835)	0.061^b^
**NRI (95% CI)**		
Category-free NRI (% [95% CI])	17% (8.9 to 25%)	< 0.001
% of events correctly reclassified	-8%	0.036
% of non-events correctly reclassified	25%	< .0001
**IDI (95% CI)**	0.002 (0.0046–0.0143)	0.046

CKD, chronic kidney disease; FLI, fatty liver index; AUC, area under the curve; CI, confidence interval; NRI, net reclassification improvement; IDI, integrated discrimination improvement

^a^ Traditional model includes age, gender, baseline eGFR, smoking, regular exercise, alcohol intake, protein intake, systolic BP, DM, total cholesterol and log hsCRP

^b^ P-value (Traditional model vs. Traditional model + FLI)

## Discussion

In our current large prospective cohort study, we found that the presence of NAFLD, as assessed by FLI, is associated with incident CKD independent of traditional risk factors (i.e., DM and hypertension) during a 10-year period. Our findings support the application of NAFLD as a risk factor in the early pathogenesis of CKD. Furthermore, we found that the addition of FLI to traditional CKD risk factors improved the prediction of incident CKD, including risk reclassification metrics. To the best of our knowledge, this is the first prospective cohort study to explore the clinical utility of FLI on risk prediction of incident CKD.

CKD is a risk factor for end-stage renal disease (ESRD) and cardiovascular disease. ESRD develops in a substantial proportion of CKD patients receiving standard-of-care therapy, and mortality in CKD remains unchanged [24]. This finding indicates that key pathogenic mechanisms underlying renal function decline are unaffected by current management and calls attention to the need for easily identifiable risk factors and novel pharmacological targets. As growing evidence supports a close relationship between NAFLD and CKD, NAFLD has been considered a risk factor for renal dysfunction. However, to date, few cohort studies have investigated the impact of NAFLD on renal dysfunction. Targher et al. reported that NAFLD diagnosed by ultrasonography was associated with development of CKD during 6.5 years in 1,760 subjects with type 2 diabetes mellitus [25]. However, subjects with diabetes mellitus are at higher risk in CKD. In addition, because that study did not consider inflammatory status (e.g., hsCRP) in analyzing the association between NAFLD and incident CKD, the authors could not clarify the independent effect of NAFLD on incident CKD. Chang et al. also demonstrated that NAFLD assessed by ultrasonography was associated with incident CKD over 3.2 years in a community-based cohort of 8,329 men [26]. However, their study did not exclude subjects who consumed alcohol heavily or received medication that affected renal function. Accordingly, we assessed whether NAFLD contributed to the development of CKD independent of other confounding factors in relatively healthy Korean adults.

In the current study, we observed that hepatic steatosis as assessed by FLI is associated with development of CKD independent of traditional risk factors for CKD. This finding confirms the deleterious role of hepatic fat accumulation in the occurrence and worsening of features of renal dysfunction. Although the pathogenic mechanisms linking NAFLD and CKD are not fully understood, the most plausible explanation for this may be the shared cardio-metabolic risk factors and similar pathogenic mechanisms such as chronic inflammation, dyslipidemia, and insulin resistance [27]. However, our study clearly demonstrated that NAFLD was associated with incident CKD after further adjustment for cardio-metabolic risk factors such as hypertension, diabetes mellitus, levels of cholesterol, and hsCRP. The independent association between NAFLD and incident CKD can be explained by following several mechanisms. Renin–angiotensin system (RAS) activation in NAFLD might lead to the development of renal dysfunction. In the liver, Angiotensin II promotes de novo lipogenesis and production of pro-inflammatory cytokines like interleukin-6 (IL-6) and tumor growth factor-β (TGF- β), triggering fibrogenesis [28–31]. In the kidney, RAS activation plays a key role in determining renal ectopic lipid deposition, which is known to cause oxidative stress and inflammation through hemodynamic effects of glomerular efferent arteriole vasoconstriction, leading to glomerulosclerosis [32]. In addition, systemic release of several coagulation factors from the steatotic liver, such as plasminogen activator inhibitor-1 (PAI-1) and fibrinogen, is also implicated in the development of CKD [33]. Finally, altered level of hepatokines (i.e. fetuin-A) from an inflammed liver can modulate inflammatory processes that in turn mediate endothelial dysfunction and consequently induce renal impairment [34].

Regarding the close and biologically plausible relationship between NAFLD and CKD, we hypothesized that presence of NAFLD might be used as a predictor of renal dysfunction in individuals. Although it is the gold standard of NAFLD diagnosis, liver biopsy is quite invasive and not routinely used in clinical practice [35]. However, FLI is a simple index which only requires measurement of BMI, waist circumference, GGT, and TG. The FLI can detect NAFLD assessed by magnetic resonance spectroscopy with considerable accuracy [14] and has been well validated in a Korean population [15,36]. Therefore, to determine the clinical utility of FLI as a predictor of renal dysfunction, we used statistical methods, including two newly described metrics, IDI and NRI. Alongside the incremental AUC, these complementary parameters may be considered new standards for evaluating incremental values of predictors [37]. Interestingly, we found that FLI showed added value in each of these metrics. Moreover, the absolute values of NRI and IDI achieved when using FLI were substantive when compared with other recognized risk factors in both the general literature and specifically in CKD [38]. Consequently, our results suggest that FLI is a simple and useful clinical predictor for CKD and can be applied to predict and prevent renal function decline.

This study has several limitations. First, although FLI is well validated surrogate marker of NAFLD in several epidemiologic studies [39,40], we did not perform histologic confirmation using liver biopsy in diagnosing NAFLD. Second, because this cohort did not collect data on proteinuria, we could not include incident proteinuria for defining incident CKD. Therefore, we may have underestimated the overall incidence of CKD. In addition, GFR was not directly measured, but was estimated using a serum creatinine-based equation that might have overestimated or underestimated the actual GFR [41]. Third, we could not collect all data in terms of the kinds of anti-hypertensive medication and usage of drugs that may affect kidney function. Finally, because our study subjects were not representative of the general Korean population, the generalization of our findings to other populations is unknown. Despite the above limitations, our study also has several strengths. First, our data were obtained from a long-term follow-up cohort study that included a large number of subjects. Furthermore, it is the first population-based prospective study to explore the potential role of NAFLD in the pathogenesis of CKD in relatively healthy general population.

Collectively, the presence of NAFLD determined by FLI substantially increased the risk of incident CKD over a 10-year period in an adult population. Furthermore, we presented that FLI provided modest to substantial improvement in reclassification of incident CKD beyond traditional risk factors. Our findings suggest that FLI is useful to better classify CKD risk in individuals. Given the increasing prevalences of both CKD and NAFLD and their direct effects on the acceleration of cardiovascular disease, further large-scale studies with stratification by the traditional CKD risk factors such as hypertension or diabetes mellitus are warranted to confirm the utility of FLI as a predictor for incident CKD.
